# Non-blood medical care in gynecologic oncology: a review and update of blood conservation management schemes

**DOI:** 10.1186/1477-7819-9-142

**Published:** 2011-11-03

**Authors:** Maria Simou, Nikolaos Thomakos, Flora Zagouri, Antonios Vlysmas, Nikolaos Akrivos, Dimitrios Zacharakis, Christos A Papadimitriou, Meletios-Athanassios Dimopoulos, Alexandros Rodolakis, Aris Antsaklis

**Affiliations:** 1 Department of Obstetrics and Gynecology, Alexandra Hospital, School of Medicine, University of Athens, Greece; 2 Department of Clinical and Therapeutics, Alexandra Hospital, School of Medicine, University of Athens, Greece

**Keywords:** bloodless surgery, gynecologic oncology, blood salvage, hemodilution

## Abstract

This review attempts to outline the alternative measures and interventions used in bloodless surgery in the field of gynecologic oncology and demonstrate their effectiveness. Nowadays, as increasingly more patients are expressing their fears concerning the potential risks accompanying allogenic transfusion of blood products, putting the theory of bloodless surgery into practice seems to gaining greater acceptance. An increasing number of institutions appear to be successfully adopting approaches that minimize blood usage for all patients treated for gynecologic malignancies. Preoperative, intraoperative and postoperative measures are required, such as optimization of red blood cell mass, adequate preoperative plan and invasive hemostatic procedures, assisting anesthetic techniques, individualization of anemia tolerance, autologous blood donation, normovolemic hemodilution, intraoperative cell salvage and pharmacologic agents for controlling blood loss. An individualised management plan of experienced personnel adopting a multidisciplinary team approach should be available to establish non-blood management strategies, and not only on demand of the patient, in the field of gynecologic oncology with the use of drugs, devices and surgical-medical techniques.

## Review

With the advent of technology and advanced procedures in the field of medicine, an emerging issue of restricting allogenic blood transfusion has arisen. The medical knowledge gained in the care of Jehovah Witnesses has turned the concept of restriction of blood transfusions into reality and redirected transfusion medicine towards a more blood conservation oriented management [1]. Bloodless surgery schemes are part of a multidisciplinary approach to patient care that involves all the measures and clinical strategies that are taken in order to prevent or at least minimise blood loss without allogenic transfusion [2,3]. Current and emerging advances have offered a new approach to the surgical management of patients that refuse an allogenic blood transfusion.

Nowadays, increasingly more patients are expressing their fears concerning the potential risks accompanying the transfusion of blood products and requesting non-blood surgical management; the potential hazardous effects of allogenic transfusion can be categorised into infectious and non-infectious risks as well as effects of immunologic etiology [4]. Implications of blood transfusion occur more often in patients treated for hematologic disorder or malignancy at a rate of 1% to 6% [5,6].

There is growing concern regarding viral contamination of blood with the human immunodeficiency virus, hepatitis B and C viruses, Ebstein-Barr virus, human T-cell lymphotropic viruses, cytomegalovirus, non A and non B hepatitis viruses; quite rare infections result from the West Nile virus and parasites such as babesiosis, Chagas disease and malaria [7,8].

Non-infectious complications of blood transfusion mainly involve transfusion errors, occurring at a rate of 1 in 12, 000 transfusions performed, with fatality rates of 1 death in 600, 000 transfusion errors [9,10], as well as circulatory overloads. Less frequent non-infectious complications include adult respiratory distress syndrome, hypothermia, hemosiderosis, arrhythmia, hypocalcemia and hypomagnesemia [4]. Among the effects of immunologic etiology, as a result of blood transfusion, are reactions of acute and delayed hemolysis, fever, allergic reactions, post-transfusion purpura and transfusion-related acute lung injury [11].

Finally, some patients, such as Jehovah Witnesses, adamantly refuse the transfusion of blood and its products on the basis of their religious beliefs, even when they are exposed to life threatening situations. Such rights of self-determination are highly respected and have driven large medical institutes to establish Bloodless Surgical Measures and Schemes [12]. Today, many centers worldwide and over 50 in the United States alone practise bloodless surgery [13].

Blood transfusion in the surgical management of gynecologic oncology patients seems to be a common approach [14]. A considerable percentage of women undergoing abdominal hysterectomy (12.4%-16.7%) need to be transfused with blood or its products [15,16]. Nevertheless, even in the field of gynecologic oncology, surgeons are obliged to comply with the patients wish for application of non-blood management strategies in order to avoid blood-borne risks associated with blood transfusion. Awareness and incorporation of such interventions is mandatory and should be followed by all surgeons and all patients have the right to benefit from the application of these measures.

### Gynecologic Oncology Surgery experience in blood management

An independent review of the literature revealed seventeen clinical studies that have examined the effect of blood conservation management schemes in patients undergoing surgery for gynecologic malignancy or major pelvic surgery in general [3,17-31] (Table 1).

**Table 1:** Clinical studies evaluating blood conservation methods in major pelvic surgery and gynecologic cancer patients

Study author/year	Number of patients	operation/pathology	Methods of blood conservation
Mays 1976[17]	51	Gyn surgery/obstet.	Iron-dextran
Bonakdar 1982[18]	164	Major gyn surgery/obstet.	No transfusion
Powell 1983[19]	26	Radical hysterectomy &pelvic lymphadenectomy	Nitroglycerine hypotensive anesthesia
Takemura 1989[20]	3	Stage III cervical adenocarcinoma	Preoperative transcatheter arterial embolisation
Eisencop 1990[21]	58	Radical hysterectomy & retroperitoneal lymph node dissection: stage IB cervical cancer	Non transfused vs transfused perioperatively
Florica 1991[22]	28	Pelvic exenteration	Albumin infusion & crystalloids postoperatively
Look 1993[23]	97	Squamous vulvar carcinoma	Non transfused vs transfused postoperatively
O'Dwyer 1993[24]	168	Abdominal hysterectomy	Autologous blood transfusion
Kelley 1994[25]	8	Extensive pelvic operations	Perioperative normovolemic hemodilution/homologous transfusion
Monk 1995[26]	134	Radical hysterectomy: stage IA2-IIA cervical cancer	Non transfused vs transfused peri/postoperatively
Connor 1995[27]	31	Radical hysterectomy for early cervical cancers	Intraoperative autologous blood collection & autotransfusion
Mirhashemi 1999[42]	50	Radical hysterectomy type III for erly cervical cancer	Intraoperative autologous blood transfusion
Stovall 2001[28]		Gynecologic cancer patients under chemotherapy	Epoetin Alpha
Dildy 2006[29]	1	hysterectomy	Pelvic pressure pack
Massiah 2006[30]	14	Major gynaecological procedures	No transfusion(Jehovah's witnesses)
Nagarsheth 2007[3]	1	leiomyosarcoma	Iron, folate, erythropoietin, uterine artery embolisation, recombinant VIIa, cell salvage, crystalloids
Nagarsheth 2009[31]	3	Leiomyosarcoma, ovarian adenocarcinoma, pelvic mass	Blood salvage

As early as 1976, Mays et al presented the infusion of iron dextran diluted in 1000 ml normal saline in 51 patients undergoing gynecologic surgery [17]. A great hemoglobin response of 1.9 gr per decilitre per week was demonstrated in this group of patients. No allergies occurred and it proved to be a safe and reliable method. Some published studies have investigated the impact of avoiding allogenic blood transfusion on the outcome of patients undergoing major pelvic operations, treated for gynecologic malignancies [18,21,23,26,30]. Bonakdar et al [18] retrospectively reviewed 164 Jehovah Witnesses undergoing major gynecologic and obstetrical interventions without blood transfusion comparing them to 164 control patients. The study added effectual evidence to the notion that major gynecologic interventions can be performed without the need of blood and its products. Eisenkop et al reported that perioperative blood transfusion adversely affected the outcome of 68 patients undergoing radical hysterectomy for cervical cancer stage IB compared to 58 patients treated the same way, but not transfused. The disease recurred in 14.7% of the transfused group, while recurrence of the disease was 3.4% in the non-transfused group (p = 0.035) [21].

The recurrence of gynecologic malignancy was not demonstrated after allogenic blood transfusion in the study of Look et al, who examined 154 patients operated for squamous vulvar cancer. He divided patients into two groups: transfusion was given to 57 patients while the remaining 96 received no blood. Both groups revealed similar disease recurrence rates [23]. These results are in line with those of Monk's study, who tried to evaluate the overall survival and time to recurrence among 131 patients transfused during radical hysterectomy for cervical cancer stage IA2-IIA and 134 patients who were offered the same operation for the same disease but were not perioperatively transfused. No difference was noted between the two groups [26]. Finally, Massiah concluded in his own study that major, intermediate and minor gynecological procedures can be successfully performed in Jehovah Witnesses. Among the 64 procedures, there were 14 major gynecological operations [30].

An effective preoperative measure to decrease perioperative blood loss and therefore minimise the need for blood transfusion is presented by Takemura et al [20]. Transcatheter arterial embolisation in three cases of cervical adenocarcinoma stage III was carried out preoperatively, in order to stop hemorrhage. The method proved to be quite effective. The same preoperative method of arterial embolisation was used by Nagarsheth et al before operating on a 52-year-old woman for a pelvic mass of 40 cm [31]. Bilateral uterine artery embolisation was attempted together with other measures to minimise blood transfusion preoperatively, such as weekly erythropoietin, iron and folate therapy. Intraoperative measures included recombinant factor VIIa and salvage of 280 ml of red blood cells. O'Dwyer described his experience on autologous blood donation preoperatively in 168 women undergoing abdominal hysterectomy; it was presented by the authors as a safe and reasonable transfusion practice [24].

Intraoperative measures for controlling blood loss and minimising allogenic blood transfusion in the field of gynecologic oncology have also been described. Powell et al presented the effect of nitroglycerine based hypovolemic general anesthesia during radical hysterectomy and pelvic node dissection in 26 patients [19]. Compared to the control group, the guided hypotension during surgery seemed to decrease blood loss by 70% and shorten operating time by 29.5%. Consequently, blood transfusion was required in a greater percentage of patients in the control group (82% vs 11.5%). Intraoperative hemodilution was attempted by Kelley and his associates, who used an extracorporeal circulation device (Haemonetics-V50 Cell Separator) in order to conserve blood during surgery in 8 women treated with extensive pelvic operations [25]. Two women accepted homologous transfusion, while the mean estimated blood loss was calculated at 75 to 2000 ml. Connor et al, on the other hand, divided 71 women undergoing radical hysterectomy for early cervical cancer into two groups. Intraoperative autologous blood collection was performed in both groups; 31 women received their own blood collected by Cell Saver and 41 women were not autotransfused. Connor concluded that intraoperative autologous blood collection decreases the need for homologous transfusion and does not facilitate co-transfusion of malignant cells^27^. Mirhashemi and his associates described the use of autologous blood transfusion in 50 women undergoing radical hysterectomy type III for early cervical cancer. There seemed to be no compromising malignancy outcome. Last but not least, Nagarsheth described the surgical removal of a 12.7 kg leiomyosarcoma without allogenic blood transfusion [31]. During the operation, recombinant factor VIIa was used together with cell salvage of 282 ml concentrated blood reinfused after filtering with a leukocyte depletion filter. Nagarsheth reported two more cases in which the same technique of cell savage was used [31]. He reinfused 400 ml of salvaged blood into a 58-year-old woman operated for ovarian adenocarcinoma and 170 ml of salvaged blood into a 49-year-old female operated on for a large pelvic mass, which proved to be a gastrointestinal stromal tumor. In all three cases, the leukocyte depletion filtering system was used. The woman suffering from the gastrointestinal tumor died of the disease one year later.

Concentrated albumin infusion has been described by Florica et al as a useful postoperative recovery tool in women who undergo pelvic exenteration [22]. Postoperatively, one group of 10 women received an albumin 25% infusion coupled with crystalloids, while 18 women received the crystalloid infusion only. The overall outcome in the albumin infusion group proved to be better in terms of stable postoperative course and length of stay in the Intensive Care Unit for those patients offered such major operations. Moreover, measures such as Epoietin Alpha and pressure pack for pelvic hemorrhage have been efficient in controlling blood loss postoperatively and decreasing allogenic transfusion requirements [28,29]. Epoietin Alpha has been associated with hemoglobin increase in gynecologic cancer patients receiving chemotherapy as a weekly dose [28].

### Principles of bloodless surgery

Gynecologic oncologists commonly deal with massive hemorrhage during major pelvic operations and quite often an emergency intervention is required to save the patient's life or deal with acute blood loss. So far, no organised plan for bloodless surgery in gynecologic oncology has been established, apart from the results presented at the 2006 International Gynecologic Cancer Society Meeting and the 2006 Society for the Advancement of Blood Management Meeting [32]. In the interest of simplicity, interventions used in bloodless surgery can be categorised into preoperative, intraoperative and postoperative measures.

#### Preoperative measures

A most important aspect in the surgical management of those gynecologic oncology patients who are hesitant to receive blood transfusions is that of appropriate preoperative counselling. Surgeons should be knowledgeable and skilled in advanced non-blood techniques; they should inform the patient of available alternatives to transfusion, discuss the risk-benefit ratio of all these measures and propose the best strategies. A specially prepared consent form that clearly outlines the necessary therapeutic options in each case and the strategies accepted by the patient should be offered preoperatively. Each and every woman is considered responsible for any decision concerning management of her health and has the right to accept or refuse an applied treatment option. Similarly, gynecologic oncologists should respect patients' beliefs and informed choices.

Previous studies have revealed anemia in a significant percentage of patients assigned to elective surgery that can vary from 5% to 75% [33,34]. The best option would be to optimise hemoglobin level before surgery and reinforce red blood cell mass formation with the administration of oral or intravenous iron, vitamin B12 or folic acid preparations. Oral iron seems to be a good choice, but quite often intravenous iron is recommended at 1 to 2 weeks intervals [4]. A hemoglobin of 13 gr/dl can be considered an acceptable goal preoperatively [35].

Another and more effective alternative for the correction of preoperative anemia is the administration of recombinant human erythropoietin (rHuEPO). Its action is mainly based on its effect on bone marrow which in turn increases red blood cell mass [36]. Nevertheless, the use of erythropoietin stimulating agents (ESA) has provoked concerns regarding safety when administered to optimise haemoglobin levels exceeding 12 gr/dl, due to thromboembolic and cardiovascular events reported [37]. Moreover, still under investigation is the use of erythropoietins in cancer patients, as such agents might act as growth factors for certain tumors [38]. FDA has therefore proposed the use of erythropoietins in anemia related to chemotherapy in oncologic patients [37].

Preoperative autologous blood donation is another alternative. This actually involves the donation of 4 units of whole blood preoperatively over a 4-week period; the blood is then stored and given to the patient, as required, with autologous transfusion [39]. Nevertheless, a limitation to autologous donation is a hemoglobin of no less than 11 gr/dl and its infectious potential [4]. Autologous blood donation may decrease the incidence of immunosuppression reported in homologous blood transfusions, in gynecologic oncology [4].

Finally, in the hands of well-trained interventional radiologists, uterine artery embolisation has been reported in the literature as an effective preoperative technique that minimises intraoperative blood loss [3,20]. Potential risks include fertility compromise, the classic post-embolisation syndrome (infection, peritoneal and intrauterine adhesions) and irradiation hazard [40].

#### Intraoperative measures

Intraoperative blood loss could be effectively minimised by meticulous hemostasis, reduction of operative time, hypotensive anesthetic techniques, intraoperative hemodilution, blood salvage and pharmacological hemostatic agents.

Hypotensive states during major pelvic surgery, using general anesthetic agents coupled with nitroglycerine, effectively minimise blood loss with mean arterial pressure reaching as low as 60 mmHg [19]. Contraindications to this method are cerebrovascular disease, severe renal and hepatic compromise, myocardial ischemia, hypovolemic status and peripheral vascular disorder [19].

Hemodiluting methods, either hypervolemic or isovolemic, are rarely utilised in the field of gynecology [4]. Hypervolemic hemodilution demands that large volumes of solutions - crystalloids or colloids - are infused in volume boluses calculated at 3 times the calculated blood loss, so as to maintain a greater amount of haemoglobin [11,14]. Greater intravascular oncotic pressure with smaller volumes can be accomplished more effectively with colloids rather than crystalloids [11]. During hypovolemic hemodilution, 1 to 2 units of whole blood are preoperatively collected and substituted with volumes of solutions. The blood can then be easily transfused back to the patient against hypovolemia [41]. Severe anemia, pregnancy and use of beta-blockers represent contraindications for hemodiluting methods [4].

Perioperative autotransfusion or blood salvage, is a technique during which blood is collected intraoperatively from the patient's abdomen or pelvis, processed through leukocyte depletion filters or irradiation measures [42,43] and then transfused back to the patient being operated [11]. Unfortunately, the use of such a method is currently restricted in cancer patients due to the potential hematogenous dissemination of malignant cells [31]; indications would be abdominal uterine myomectomy, ectopic pregnancy operations and abdominal hysterectomy for benign disease [44,45]. Nevertheless, studies in the literature have shown that the use of blood salvage on gynaecological oncology patients poses no such risk [27,31,42]. Potential risks accompanying the method are fat and air embolism and infection [4].

Aminocaproic acid, desmopressin acetate, aprotinin, tranexamic acid, phytonadione and vasopressin are hemostatic drugs also utilised for the control of intraoperative hemorrhage [44]. Aprotinin exerts antifibrinolytic and anti-inflammatory action; though usually preferred over the other agents, it often causes thromboembolic sequelae and renal compromise, and is quite costly [46]. Additionally, recombinant factor VIIa (rFVIIa) has contributed to a great reduction in blood usage, even in the field of gynecologic oncology, although its use in managing perioperative coagulopathy is 'off-labelled'^3^. Intraoperatively, the use of rFVIIa may provoke thromboembolic events at a rate of 44% [46,47]. Hence, pharmacologic hemostatic agents should be applied with caution and not to all cases of intraoperative bleeding.

#### Postoperative measures

Postoperative measures include meticulous postoperative monitoring of the patient, early recognition of blood loss [39,47], minimisation of phlebotomy blood sampling [39], enhancement of hemopoiesis [45], optimisation of cardiopulmonary status [48] and minimisation of oxygen consumption to provide adequate perfusion to tissues [14]. Albumin may be continuously infused by gynecologic oncologists early on postoperatively in order to stabilise blood pressure and establish fluid load [22]. Epoietin Alpha (Epo) can be used in anemic cancer patients under chemotherapy [49]; similarly, Granulocyte-macrophage colony-stimulating factors and platelet growth factor could be considered in the treatment of chemotherapy-induced thrombocytopenia in women suffering from gynecologic malignancies [4].

## Conclusion

In the field of gynecologic oncology, the perioperative management of patients who refuse allogenic blood transfusion, poses limitations for surgeons and renders mandatory the establishment of Bloodless Surgery Programs; each gynecologist should be informed about the available blood conservation methods and order their application if needed, optimising the patients' outcome without allogenic blood transfusion. Such actions must be initiated by a multidisciplinary approach with the coordination of all members of the bloodless medicine and surgery team such as surgeons, anaesthesiologists, intensivists, pharmacists, nursing stuff and hematologists. The efficient cooperation of all members of the team will guide institutions towards a marked blood usage reduction over time.

## Conflict of interest

The authors declare that they have no competing interests.

## Authors' contributions

MS: conceived the idea, assisted in writing the manuscript; NT: conceived the idea, assisted in writing the manuscript, made MEDLINE research; FZ: assisted in writing the manuscript, made MEDLINE research, submitted the manuscript; AV: made MEDLINE research; NA: made MEDLINE research; DZ: made MEDLINE research; CP: made revisions in the final version of the manuscript, gave final approval for manuscript submission; MAD: made revisions in the final version of the manuscript, gave final approval for manuscript submission; AR: made revisions in the final version of the manuscript, gave final approval for manuscript submission; AA: conceived the idea, made revisions in the final version of the manuscript, gave final approval for manuscript submission.
